# An Emerging Role of m6A in Memory: A Case for Translational Priming

**DOI:** 10.3390/ijms21207447

**Published:** 2020-10-09

**Authors:** Amanda M. Leonetti, Ming Yin Chu, Fiona O. Ramnaraign, Samuel Holm, Brandon J. Walters

**Affiliations:** 1 Department of Biology, University of Toronto Mississauga, Mississauga, ON M5S 3G5, Canada; amanda.leonetti@mail.utoronto.ca (A.M.L.); mingyin.chu@mail.utoronto.ca (M.Y.C.); fiona.ramnaraign@mail.utoronto.ca (F.O.R.); 2 Department of Psychology, University of Toronto, Mississauga, ON M5S 3G5, Canada; 3 Department of Cell and Systems Biology, University of Toronto, Mississauga, ON M5S 3G5, Canada; samuel.holm@mail.utoronto.ca

**Keywords:** m6a, FTO, memory, behavior, epitranscriptomic, epigenetic, mettl, ythdf

## Abstract

Investigation into the role of methylation of the adenosine base (m6A) of RNA has only recently begun, but it quickly became apparent that m6A is able to control and fine-tune many aspects of mRNA, from splicing to translation. The ability of m6A to regulate translation distally, away from traditional sites near the nucleus, quickly caught the eye of neuroscientists because of implications for selective protein translation at synapses. Work in the brain has demonstrated how m6A is functionally required for many neuronal functions, but two in particular are covered at length here: The role of m6A in 1) neuron development; and 2) memory formation. The purpose of this review is not to cover all data about m6A in the brain. Instead, this review will focus on connecting mechanisms of m6A function in neuron development, with m6A’s known function in memory formation. We will introduce the concept of “translational priming” and discuss how current data fit into this model, then speculate how m6A-mediated translational priming during memory consolidation can regulate learning and memory locally at the synapse.

## 1. Introduction

Memory formation can be broadly viewed as a synaptic process that requires local changes within the synapse to support stable changes in synaptic function. These changes are initially supported by local translation induced by a learning event, but long-term changes in synaptic function require de novo nuclear transcription [1]. The combination of local molecular events at the synapse and distal events in the nucleus work in concert to support the formation of long-lasting memories. While learning-induced signaling from the synapse to the nucleus has been widely studied, it is still unclear how local changes in the synaptic proteome are regulated during learning. Here, we propose that modifications of RNA, particularly adenosine methylation, is a novel process regulating local translation of mRNA induced by learning.

N6 Methyl-adenosine (m6A) is a post-transcriptional modification of RNA in which a methyl group is installed at the 6′ carbon in adenosine. m6A is the most prevalent type of epitranscriptomic modification (hence why it is often referred to generically as ‘RNA methylation’), averaging 3 methyl groups per mRNA [2]. Since the initial in vitro characterization of m6A in 1974 [3], little progress was made toward understanding its function until recent work reignited an interest in this RNA modification. Although m6A has been implicated in regulating non-coding RNA [4], its role in mRNA regulation is better understood and as such, m6A in mRNA will be the focus of the current review. Emerging consensus is that m6A can influence nearly every fate of the tagged mRNA across complex systems [4], including the brain [5], but that precise functions of m6A depend on the spatial location it occupies within the mRNA, as well as the specific reader proteins that recognize m6A. Studies of m6A in the brain are revealing many similarities with other systems, but growing evidence is also uncovering unique uses for m6A, particularly in the synapse. This review will focus on m6A function in the brain, highlighting novel roles for m6A in the nervous system, and speculating about the function of m6A in the synapse.

## 2. The Readers, Writers, and Erasers of m6A

Epitranscriptomic proteins fall into three broad categories according to their function: readers, writers, and erasers, which regulate the function, addition, and removal of m6A, respectively. The catalytic ‘writer’ of m6A, named the methyltransferase complex (MTC), canonically consists of two proteins, METTL3 (methyltransferase-like protein 3) and METTL14 (methyltransferase-like protein 14) [6,7,8]. The MTC does not appear to function on its own, but instead associates with a wide range of accessory proteins [7,9] to form a holoenzyme that adds m6A in a co-transcriptional manner [10]. Although MTC members have been found in various cellular compartments, m6A deposition has not been described outside of the nucleus [11,12]. It is unknown if MTC members that reside outside of the nucleus have a novel function or can add m6A in a transcriptionally independent manner.

Similar to m6A writers, the dynamic removal of m6A has been described as a nuclear process [13], with eraser proteins residing primarily within nuclear speckles [13,14]. However, recent work has challenged this idea, suggesting that demethylation of m6A may not be as spatially limited as previously thought [5,11,15]. Two canonical m6A demethylases have been described: ALKBH5 (ALK B Homolog 5) and FTO (Fat Mass and Obesity-associated) [13,16,17]. FTO and ALKBH5 are abundant in the nuclear speckles and cytoplasm [14,18], but evidence suggests that FTO may have a broader distribution in neurons, including in axons [19] and dendrites [11,15], in addition to canonical localization within the nucleus and cytoplasm [15,18,20]. Debate about m6A erasers is not limited to their localization, but the true dynamics of m6A removal are also in question [5,13], with some work suggesting that the function of FTO and ALKBH5 may be more complex than originally thought [15,16,17,21].

Finally, m6A reader proteins are a class of RNA binding proteins that bind m6A-containing transcripts [22]. The best-known members of this class are the YTH-domain family of proteins, (YTHDF1-3 [23,24], YTHDC1-2 [25,26]) that bind m6A and actively direct the fate of the mRNA. Reader proteins have a diffused subcellular localization, but some members appear to be exclusive to the nucleus [25,26], while others are reported distally in dendritic/axonal regions of neurons [11], where their precise function is unclear.

## 3. Effects of m6A on mRNA Dynamics

### 3.1. Effect of m6A: Pro-Translation

m6A can be found throughout mRNA [23,27], with a bias for the stop codon and 3′ un-translated region (UTR) [2]. The outcome of m6A deposition depends both on its localization in mRNA and the specific reader protein that is recruited [16]. Despite all the complexities of m6A, there are some generalities that can be described. Work by Zhou and colleagues demonstrates that in-vitro m6A deposition within the 5′ UTR of *Hsp70* is normally removed by FTO [28]. However, in response to heat-shock, YTHDF2 re-locates to the nucleus, binds the m6A of *Hsp70*, thus preventing its demethylation [28] and promoting rapid translation of *Hsp70* through cap-independent translation [28,29]. This contrasts with m6A deposition in the 3′ UTR, which can promote cap-dependent translation by increasing the rate of translation initiation [30]. In this example, YTHDF1 recognizes m6A in the 3′ UTR and traffics the m6A-marked transcript to the ribosome, where it binds directly to the translation machinery to accelerate translation initiation [30]. Ultimately, the location of m6A on a transcript and the identity of the reader protein engaged radically changes the fate of the mRNA in question.

### 3.2. Outcomes of m6A: Pro-Degradation

mRNA degradation is a highly regulated process that modulates translation by actively removing available mRNA. Unlike its function in the 5′ UTR, binding of YTHDF2 to m6A in the coding sequence (CDS) or the 3′ UTR actively promotes degradation of the target mRNA [24]. In-vitro, both *SON* and *CREBBP* have m6A installed in the CDS or 3′ UTR of each mRNA, respectively [30]. In these examples, loss of YTHDF2 protects those transcripts from degradation and re-distributes both transcripts to the translatable pool away from the processing bodies (p-bodies) [31], where degradation is carried out. When present, YTHDF2 promotes the direct binding of the mRNA to the CCR4-NOT complex to rapidly deadenylate and degrade the transcript [32]. Thus, YTHDF2 exemplifies the flexibility and complexity of m6A-related outcomes, whereby m6A can promote or inhibit translation, depending on which transcript is recognized and where in the transcript the m6A was localized.

### 3.3. Outcomes of m6A: Splicing

While effects of m6A on mRNA half-life and translation are the focus of many studies, m6A function is in no way limited to mRNA stability. Notably, m6A is also instrumental in specific splicing events. For example, studies in *Drosophila* show that sex determination involves sex-specific editing of the Sex lethal (*sxl*) transcript in females [33]. Specifically, *sxl* pre-mRNA in females has m6A tags flanking a male-specific exon, whereby the flanking arrangement of m6A suppresses the inclusion of the flanked exon and effectively prevents its inclusion in the protein product in females [33]. Depletion of m6A via knockdown of the MTC member METTL3 resulted in misexpression of the male *sxl* isoforms in females, and produced female flies that were non-viable and expressed male sexual features, such as sex combs (Table 1) [33]. Demonstrating a role of m6A in splicing has proven elusive in mammals [34], but evidence from mammalian in-vitro studies [35], as well as observations of m6A clustering around splice junctions [36], suggests that a role for m6A in mammalian splicing may still be found.

### 3.4. Outcomes of m6A: mRNA Localization

m6A function is not limited to translation/splicing, but spatial localization of mRNA in cellular compartments can also be regulated by m6A. Roundtree and colleagues examined how m6A regulates mRNA localization from the nucleus to cytoplasm [26]. They found that the m6A reader YTHDC1 recognizes both m6A and the mRNA export machinery (specifically SRSF3) to facilitate the nuclear export of m6A-tagged mRNA [26]. This is antagonized by nuclear ALKBH5, which actively removes m6A and suppresses export of the mRNA [17]. A similar mechanism exists for mRNA degradation, where mRNA is physically moved into p-bodies for degradation in conditions when YTHDF2 reads the appropriate m6A tag [37]. This evidence illustrates that m6A effects are not limited to design or stability of mRNA but can also include the spatial distribution of transcripts within a cell.

### 3.5. Reader-Less Roles of m6A

The canonical view that m6A requires a reader protein to exert its function has largely been borne out of experimental data, but recent work has started to challenge the idea that readers are always needed, thus generating the concept of an ‘m6A switch’. Here, the addition of the m6A by itself produces a functional outcome, with no need of a reader protein to carry out the function. In support, work has demonstrated how hairpins in mRNA can be modulated by m6A at important adenosines [35]. For example, hairpin formation sterically blocks access to mRNA regions that contain runs of uracils within the hairpin, which are recognized by ribonuclear proteins and processed for translation [35]. If m6A is installed at the appropriate adenosine, the hairpin falls apart and the run of uracil’s are revealed, and translation can be carried out. Next generation sequencing techniques (PAR-CLIP) revealed that approximately 7% of all binding sites of ribonuclear binding protein HNRNPC in HEK293 cells are occupied by an m6A switch, suggesting this novel function may be more widespread than appreciated. While this research is still new, work outside of model systems will be vital to understanding the reader-less functions of m6A.

## 4. Role of m6A in the Mammalian Brain

The renewed interest in m6A has yielded a wide range of fundamental biological processes and diseases controlled by m6A including, metabolism [38], circadian rhythms [39], cancer [4], viral replication [40], and axon regeneration [41,42]. The purpose of this review is not to cover all the processes and diseases involving m6A, as many of these have been covered prior [5,43,44,45]. Instead, we focus on how m6A influences brain development and memory formation, and we briefly touch on two examples of m6A dysregulation in a diseased or damaged brain.

### 4.1. Role of m6A in the Mammalian Brain: Development

Development of the mammalian brain relies critically on the precise spatial and temporal control of gene expression [46,47] so it is not surprising that m6A, which is a critical regulator of translation, is also crucial for brain development. m6A is vital during stem cell proliferation, with METTL3 depletion reducing differentiation of embryonic stem cells (ESCs) [48]. Specifically, METTL3 depletion resulted in sustained expression of the pluripotency regulator *nanog* after ESCs differentiation began, and *nanog* expression would normally be absent. This caused a failure of differentiation of multiple lineages, including the differentiation of neurons (Table 1) [48]. Similar phenotypes were also found in neuronal precursor cells (NPCs), METTL14 depletion in the mouse cortex produced a defect in proliferation and differentiation of NPC (Table 1) [49]. This deficit was associated with premature expression of pro-differentiation genes in neural progenitor cells during active proliferation, which promoted early differentiation and incorrect layering of neurons in the brain [49]. This observation is not unique to embryonic development, as METTL3 depletion in adult NPCs also impaired their proliferation and reduced the production of new neurons (Table 1) [50]. Thus, m6A appears to be a vital regulator of proliferation and differentiation of NPCs both during embryonic development, and in specialized niches in adult animals.

### 4.2. Role of m6A in the Mammalian Brain: Memory

Memory formation relies critically on nascent transcription and translation, whereby inhibition of either process blocks the development of long-term memories [51,52]. As such, m6A provides a conceptual link between transcription and translation as key processes in memory formation. Although initial studies have demonstrated a role for m6A in memory, many mechanistic questions about m6A’s function remain. In the remainder of the review, we discuss what is currently known about m6A in memory and speculate on its functions in synaptic regulation.

#### 4.2.1. Reducing m6A Impairs Memory

Next generation sequencing studies have clearly demonstrated the widespread abundance of m6A [2]. Studies of genes whose expression is rapidly induced during memory formation show that m6A is dynamically regulated during learning. Specifically, m6A has been found on the memory-related immediate early genes (IEGs) *Arc* and *cFos*, whereby the presence of m6A promotes their translation [53]. Here, METTL3 depletion in the mouse hippocampus reduced the translation of these transcripts, and produced deficits in synaptic plasticity and memory, as measured by Morris water maze and contextual fear conditioning (Table 1) [53]. This does not stem from an inability to learn, as the mice can still learn the task with additional training stimuli (additional shocks in fear conditioning or after additional training in water maze) [53], suggesting that m6A functions selectively during memory consolidation by promoting translation of pro-memory transcripts such as *Arc* and *cFos*. Of note, a study utilizing conditional METTL3 knockout mice showed no changes in memory when measured on tone-fear conditioning task [5,54], suggesting that the contribution of METTL3 to memory formation is more nuanced than appreciated.

Further support for m6A comes from studies of m6A readers, which should conceptually mimic the depletion of the writers in memory. Indeed, YTHDF1 depletion impairs memory and synaptic plasticity that can be rescued upon re-expression of YTHDF1 [55], indicating that m6A supports memory formation by regulating translation of tagged transcripts [55]. Additionally, loss of YTHDF1 caused an overall weakening of the synapse, as evidenced by a deficit in post-synaptic density and glutamate receptor abundance, changes in spine length, and an overall decrease in miniature excitatory post synaptic currents. Indicating m6A plays an instrumental role in regulating structural changes at synapses. Interestingly, these synaptic phenotypes are similar for YTHDF1 or METTL3 depletion (Table 1) [55], suggesting a common role for both reader and writer proteins in the synapse. Combined, these studies suggest that reader proteins promote local translation of m6A transcripts in the synaptic compartment during memory consolidation and that this process may be vital for establishing long-lasting memories. Although local translation of m6A transcripts during memory formation has not been directly investigated, some studies have started to shed light on local translation by the m6A pathway in the synapse [11,19].

#### 4.2.2. FTO Depletion Enhanced Memory Formation

**Figure 1:**
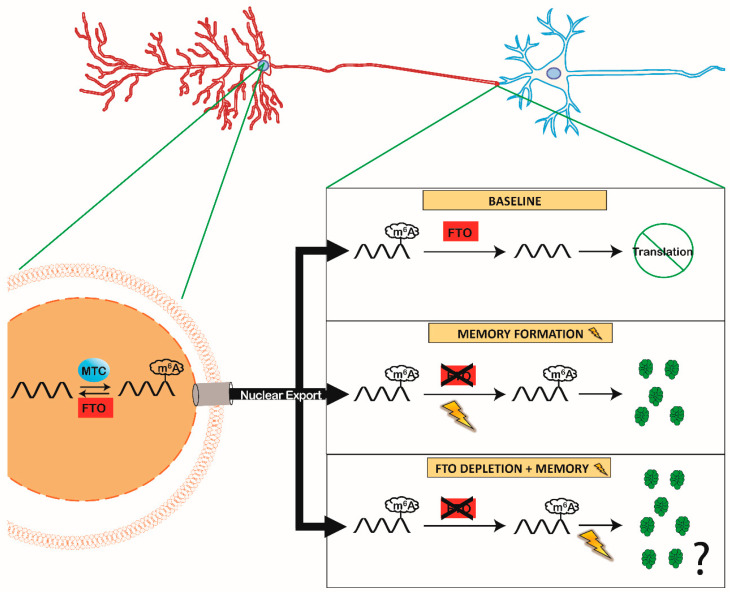
The Role of Synaptic Fat Mass and Obesity-associated (FTO) During Memory Formation. MTC members (METTL3 and METTL14) and FTO are localized within the nucleus (left) to dynamically add and remove m6A in mRNA, respectively. Under baseline conditions, this creates a competition between the methylated or demethylated status of mRNA that is shipped from the nucleus to the synapse. However, FTO is also present in the synaptic compartment [11,15,19], right) removing m6A from mRNA at baseline conditions, preventing mRNA translation. During memory formation (lightning bolt) synaptic FTO is removed inhibiting demethylation in the synaptic compartment (right middle). Resulting in a preserved m6A tag in mRNA localized at the synapse and leading to rapid translation during memory formation. Experimental evidence has shown improvement in memory when FTO is depleted ([15,56,58] right bottom), we propose that early FTO depletion increases the amount of translated pro-memory proteins in response to memory.

In addition to methyltransferase activity, m6A demethylation is also vital for memory formation. Memory formation causes a short-term reduction in the abundance of the RNA demethylase *Fto* [15,56], particularly at the synapse [15]. FTO depletion (using either CRISPR/Cas9 [15] or shRNA [15,54,56]) before training resulted in enhanced memory on the contextual fear conditioning task, consistent with results of the METTL3/YTHDF1 studies [55]. Interestingly, m6A appears to have ‘dose’-dependent effects on memory, whereby increasing m6A (by removing FTO) improves memory and too little m6A (by removing METTL3 or YTHDF1) impairs memory formation [55]. It is unclear how widespread this function of FTO is, as use of a novel FTO antagonist found that acute, systemic application in rats did not produce any changes in memory formation on the water maze task [57]. It is unclear how relatable this pharmacological finding is to other studies due to the transient and reversible nature of drug treatment compared to the permanent nature of FTO deletion. In addition, the antagonist application was designed to test traumatic brain injury (TBI) recovery and the timing of the antagonism was matched with TBI and not with the memory task [57], thus necessitating further research on the potential therapeutic benefits of FTO inhibition (diagrammed in Figure 1).

### 4.3. Role of m6A in the Mammalian Brain: Neurodegenerative Disease

#### 4.3.1. Neurodegenerative Diseases and m6A

Parkinson’s disease (PD) is an age-related neurodegenerative disease (NDD) characterized with the early death of dopaminergic neurons (DN) in the substantia nigra pars compacta, as well as other selected neurons across the brain [59]. Many PD symptoms stem from loss of these DNs and the resulting reduction in dopamine levels in the striatum [59,60] and are characterized by the development of a tremor [59] and increased risk-taking behavior [61]. Initial work in human GWAS studies identified a relationship between PD and m6A in human populations [62], which led to a search for a functional connection between m6A and PD. Initial work examining a rat model of PD (6-OHDA induced lesions of the substantia nigra) found that m6A abundance is decreased and FTO abundance is increased in the PD model compared to wild type rats [63]. The authors then used in vitro models of PD cell death (PC12 cells treated with cytotoxic insults) and found that overexpression of FTO sensitized the cells to the insults promoting apoptosis. Additionally, m6A dysregulation via FTO knockout results in abnormal reward learning [20], another key feature of PD. At this stage, work mechanistically connecting m6A with PD is still ongoing, but given the role of m6A in regulating the reward system [20], the function of dopaminergic receptors [20,63], and the survival of cells in PD models [50]. It is possible that m6A might account for both the cell death and eventual movement disorders seen in PD, but also the changes in risk-seeking behavior observed in some PD patients, although this remains to be experimentally addressed. To date, studies in PD are the clearest example of m6A function in NDDs, but additional research is beginning to shed light on a possible link between m6A and Alzheimer’s disease [64], suggesting m6A dysregulation may be a common feature of many NDDs.

#### 4.3.2. Role of m6A in the Mammalian Brain: Traumatic Brain Injury

TBI is a common devastating injury stemming from direct mechanical impacts to the brain that physically damage neurons [57]. Neuron repair relies heavily on de novo transcription and translation [65], but little work has explored m6A’s function in repair processes linked to TBI. Yu and colleagues observed m6A dynamics in response to TBI in separate brain regions and observed that *Fto* and *Mettl14* are downregulated in the rat cortex after TBI [57], but it was unclear if this was causative or part of the natural TBI response. *Fto* inhibition with a small molecule antagonist 15 min after TBI in the rat cortex exacerbated the damage seen from TBI as measured by the neurological severity score (a battery of 10 TBI sensitive tasks that measure balance, landing, and various reflexes) compared to TBI alone [57]. Surprisingly, this did not affect the memory impairments caused by TBI [57]. Work in m6A function in TBI is still in its infancy, but it will be of interest to tease apart the role of m6A after TBI in protection vs. recovery and manifestation of TBI symptoms.

## 5. Translational Priming and m6A

### 5.1. Epigenetic Priming

Epigenetic priming describes a general phenomenon whereby genes that are either silent or expressed at a low level are primed/ready to respond to an appropriate stimulus by converting a locus from a closed conformation (repressing transcription) to an open conformation [66,67,68]. While the view of ‘open vs closed’ states is still a vital mechanism of epigenetic priming, work diagramming ‘poised’ or ‘bivalent’ promoters have provided an easier framework to understand epigenetic priming. Bivalent promoters are promoters that contain both a positive and repressive transcriptional mark on the appropriate histone at the same time [69]. Although these genes are turned ‘off’, the promoter resolves into an active state in response to an appropriate stimulus. If the promoter is resolved in the positive direction, the locus starts transcription faster than if it was not primed initially [69]. While the purpose of this review is not epigenetic priming, the concept of priming is vital to our understanding of translational priming. A primed system is one that is setup for rapid activation after an appropriate stimulus. To use an automotive analogy, the system has its feet on the gas and the breaks at the same time.

### 5.2. Translational Priming

In contrast to the well-accepted epigenetic priming model, priming at the translational level has not been previously described, although some recent evidence suggests that it may be a viable mechanism [49]. As previously discussed, Yoon and colleagues demonstrate that under normal developmental conditions, certain pro-differentiation genes are prematurely expressed, but the transcripts are rapidly marked with m6A and degraded to prevent early translation [49]. When neural precursor cells are prevented from adding m6A, these transcripts become precociously translated in NPCs that are still actively dividing, thus causing early and inappropriate neuronal differentiation [49]. We speculate that the early transcription of differentiation genes coupled with their rapid degradation prior to translation bears the signs of a primed process, i.e., they have one foot on the gas (early transcription) and the other on the breaks (degradation of the early transcripts) simultaneously. Specifically, we argue that this process is consistent with differentiation genes getting ready for rapid and abundant translation when the proliferation window closes, and differentiation begins. While we do not have experimental evidence for this function, it is easy to speculate that one needs precise timing of the expression of differentiation proteins to undergo differentiation successfully, and that translational priming would fit this mechanism.

**Figure 2:**
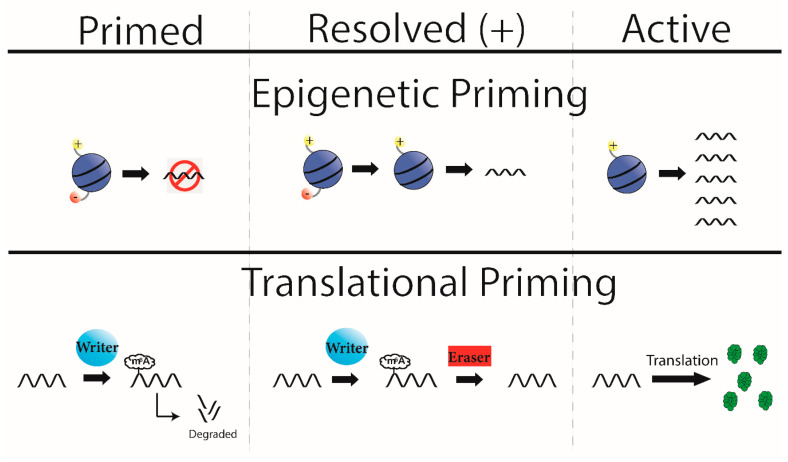
Epigenetic and Translational Priming. Bivalent histones (top) are a mechanism of epigenetic priming where a locus contains both a positive and negative transcriptional mark (left, top). At baseline this yields no or low levels of transcription, but if the appropriate signal is sensed, the bivalent domain is resolved (middle, top) here in the positive direction. This allows future stimuli to promote transcription from this locus, in a more rapid way than if it did not start with bivalent histones (right, top). Here we propose a model of translational priming that follows the same flow as epigenetic priming. Initially a transcript is primed (left, bottom), where a transcript is produced before it is needed for translation but is marked by m6A to degrade the transcript. This can be resolved by the appropriate stimuli, which removes the m6A mark (middle, bottom). The transcript can now be translated without first needing to start transcription.

Observations supporting the idea of translational priming are not limited to the nervous system, as we see a similar mechanisms in-vitro under heat shock. As previously discussed, this mechanism results from increased m6A methylation in the 5′ UTR on *Hsp70* mRNA, followed by rapid cap-independent translation, which can be mimicked by FTO depletion [28]. This suggests that under normal conditions, *Hsp70* has an m6A added in the 5′, which is quickly removed by FTO. If FTO is absent (though direct depletion or heat shock) the m6A is spared on *Hsp70* and instructs rapid cap-independent translation. The system by default installs m6A just to remove it shortly after, a similar design as prior. The foot on the gas (m6A mark for special translation) and break (removing the m6A mark). Implying that translational priming may be a widespread mechanism of m6A (diagrammed in Figure 2).

### 5.3. Potential for Translational Priming and Memory

Memory formation requires precise mRNA expression locally at the synapse, and IEGs such as *Arc* have been shown to be rapidly expressed and translated at the synapse in response to a memory task [1]. Though data demonstrating a role for translational priming for IEGs is absent, we know that the IEGs *Arc* and *cFos* are regulated by m6A expression [53]. Thus, one could speculate that *Arc,* or a similar IEG, is present in the synapse with an m6A tag, which marks the *Arc* mRNA for translation. Because there is no evidence for m6A installation outside of the nucleus, the methylated-*Arc* is then packaged and shipped locally to the synapse. At baseline, synaptically localized FTO removes the m6A tag, thus preventing *Arc* translation, either by promoting transcript degradation or by sequestration away from the translational machinery in the synapse upon m6A removal. Given that learning reduces FTO levels in the synapse (similar to what we see in [15]), this process should spare m6A on *Arc* mRNA and thus promote its translation to support memory consolidation. Thus, artificial FTO depletion would result in impaired FTO-mediated m6A removal of *Arc* mRNA, allowing it to be translated and to promote memory. Although speculative, these steps provide a testable mechanism for observed improvement in memory produced by FTO depletion.

Another intriguing theory is that certain transcripts may simultaneously contain two functionally opposed m6A marks, one signaling for translation, and the other for degradation, thus forming a bivalent mRNA that can be resolved by memory formation. In this scenario, basal FTO is functioning to demethylate only one m6A tag to resolve the ‘bivalent’ mRNA into a pure degradation signal. Upon exposure to a learning event, synaptic FTO levels are reduced, thereby sparing the bivalent mRNA. However, it is unclear what would cause the bivalent mRNA to resolve into a ‘pro-translation’ signal in this scenario. While it is intriguing to speculate, no evidence currently exists that suggests mRNA can be in a bivalent state.

## 6. Conclusions

Since the renewed interest in m6A has gained momentum, research has quickly demonstrated the importance of this system, with evidence pointing to particularly underappreciated roles of m6A in the central nervous system. Here, we put forward the speculative concept of translational priming in the brain that is informed by data in differentiating cells [49]. If this concept stands up to experimental rigor, this novel mechanism has extensive potential to fine-tune local synaptic translation and thus provide a critical missing link between cell-wide transcriptional changes in the nucleus and synapse-specific translation needed for memory formation.

## Figures and Tables

**Table 1:** Summary of Outcomes of methyltransferase complex (MTC) depletion.

Depletion	Organism	Phenotype	Source
METTL3	*Drosophilia*	Misexpression of male sex features	[33]
METTL3	Mouse ESCs	Sustained expression of pluripotency regulators and failed differentiation	[48]
	Mouse NPCs		[50]
METTL14	Mouse NPCs	Failed proliferation and differentiation	[49]
METTL3	Mouse hippocampus	Reduces IEG translation and impairs synaptic plasticity and learning	[53]
METTL3	Mouse hippocampus	Impairs structural changes at the synapse during memory	[55]
